# High-Frequency Language Therapy with Semantic Feature Analysis (SFA) and Transcranial Direct Current Stimulation (tDCS): A Longitudinal Single-Case Report of Semantic Variant of Primary Progressive Aphasia (svPPA)

**DOI:** 10.3390/brainsci14020133

**Published:** 2024-01-27

**Authors:** Katharina Strunk, Sabine Weiss, Horst M. Müller

**Affiliations:** 1 Experimental Neurolinguistics Group, Bielefeld University, Universitätsstrasse 25, 33615 Bielefeld, Germany; sabine.weiss@uni-bielefeld.de (S.W.); horst.mueller@uni-bielefeld.de (H.M.M.); 2 Center for Cognitive Interaction Technology (CITEC), Bielefeld University, Inspiration 1, 33619 Bielefeld, Germany; 3 Clinical Linguistics, Bielefeld University, Universitätsstrasse 25, 33615 Bielefeld, Germany

**Keywords:** semantic variant of primary progressive aphasia (svPPA), semantic feature analysis (SFA), transcranial direct current stimulation (tDCS), language

## Abstract

Background: The goal of this study was to investigate whether the combination of semantic feature analysis (SFA) and transcranial direct current stimulation (tDCS) is effective in treating word retrieval in the semantic variant of primary progressive aphasia (svPPA) and how long the potential effects last. Methods: A 56-year-old woman diagnosed with frontotemporal dementia (FTD) and svPPA participated in this longitudinal single-subject design. A total of four 2-week stimulation phases were conducted over a 14-month period, each of which was started depending on the participant’s language performance. Follow-up testing was conducted shortly after the stimulation period, approximately 2 weeks, and approximately 4 weeks thereafter. Results: Significant improvement in word retrieval occurred after SFA and tDCS therapy. Two weeks after the end of each stimulation phase, approx. 80% of the trained words could be named correctly. For the untrained words, also significantly more words were correctly named at follow-ups compared to the baseline. Furthermore, the Boston Naming Test (BNT) demonstrated a significant increase in naming performance and showed that phonological cues facilitated word retrieval compared to semantic cues. Conclusion: The combination of SFA and tDCS was able to counteract the expected language deterioration of a participant with svPPA. This effect increased until approximately 2 weeks after each intervention. In addition, a generalization of the effect to untrained words was shown.

## 1. Introduction

Frontotemporal dementia (FTD) belongs to the neurodegenerative dementias and is characterized by progressive deficits in behavior, executive functions, and language. A distinction is made between the frontal or behavioral variant of FTD (bvFTD) and primary progressive aphasia (PPA). PPA can be further subdivided into the non-fluent agrammatic variant (nfvPPA), the logopenic variant (lvPPA), and the semantic variant (svPPA) (for reviews see [1,2,3]).

The semantic variant of PPA (svPPA) is characterized primarily by language impairment at the level of single-word comprehension and word retrieval, especially for low-frequency words. Surface dyslexia and dysgraphia can often be observed. Intact repetition and articulation with concomitant already pronounced semantic deficits are other features of svPPA. Speech production is usually grammatically correct and only isolated paragrammatical errors occur [1]. Semantic knowledge is increasingly impaired, as reflected in impaired recognition of objects and faces. The semantic deficits are evident for all categories of concepts (animals, fruits, etc.) and modalities. In addition, early onset behavioral changes such as loss of empathy and the development of compulsions may occur [1,3,4]. Other language domains usually remain relatively intact, at least at the early stages of the disease [1].

To counteract the degradation of semantic memory and conceptual knowledge, several promising therapeutic procedures have been developed and tested (for an overview see [5,6]). Due to the common prevalence of naming difficulties, the focus is on the study and therapy of lexical retrieval (for an overview see [7,8]). In general, therapeutic interventions to improve lexical semantics are based on either semantic (e.g., description of perceptual features, purpose) or phonological (e.g., initial phoneme or syllable) approaches. However, there are also combined or multimodal approaches [9,10,11,12,13]. Other therapeutic interventions aim at promoting strategies of self-control and self-directed cueing by asking the patient to activate residual semantic and phonematic but also orthographic knowledge about words that cannot be initially retrieved [5,14,15]. A recent comprehensive review found that methods for treating lexical retrieval and improving functional communication strategies are at the forefront of current practice guidelines. Moreover, lexical retrieval treatment appears to be particularly effective in the early stage of svPPA [16].

A well-known and successful approach in this context is semantic feature analysis (SFA), which is suitable for the therapeutic activation of conceptual knowledge loss. The semantic feature approach is based on the fact that parts of the distributed semantic networks still function initially [17]. The goal of SFA is to systematically activate semantic networks so that semantic features and concepts can be linked in a structured way, facilitating access to the meaning of a concept [18]. Here, “concept” is understood as an organized structure of semantic features that confer meaning. Accordingly, a concept can have different semantic features [18]. The structured procedure of SFA is also suitable for further transfer to spontaneous speech and should be used consistently to promote communicative skills [19]. Studies that used SFA or a semantic-based therapy to treat svPPA patients almost always achieved improvements in naming trained items, whereas generalization to untrained items was not guaranteed (for extensive overviews see [5,6,20]). Thus, semantic knowledge of trained items seems to last even several months whereas near transfer to untrained items and far transfer to communicative skills in everyday life occur less frequently and are, therefore, often not found [6] (for a review see [5]). However, near transfer to untrained items is more likely in milder cases of svPPA as long as semantic knowledge is available to support word retrieval [7,21]. The question of how long improvement in naming ability can be achieved by therapeutic intervention has been investigated in some studies [20]. Sustained improvement has been observed between one and twelve months [5]. However, these effects diminish over time. For example, after three to six months, only 10–65% of trained words could be named correctly [6].

Some recent studies show that the short-term and long-term effects of semantic therapy are enhanced and supported when non-invasive brain stimulation is performed simultaneously. Notably, the generalization of therapeutic effects to untrained items appears to be more frequent and stable (for a review see [20]). For instance, in the therapeutic intervention of PPA, comparatively inexpensive and safe transcranial direct current stimulation (tDCS) showed promising results (for overviews see [20,22,23,24]). Here, anodal tDCS increases the excitability of cortical neurons while cathodal simulation decreases it [25]. This may enhance the effect of speech and language therapies (for recent reviews see [16,20,24,26,27]).

The effect of tDCS on language processing was demonstrated in a larger group of 12 svPPA patients [28]. Subjects performed a computerized semantic matching task following a double-blind, sham-controlled, balanced crossover design. However, they performed this task only after the electrical stimulation. The results showed that offline anodal stimulation of the left anterior temporal lobe led to an improvement in semantic accuracy. These results suggest a specific functional improvement within the semantic system by tDCS compared to sham stimulation in svPPA patients, which has also been confirmed in another study [28,29]. A further study investigated picture naming abilities with anodal tDCS/sham stimulation to the left inferior parieto-temporal region in two individuals with svPPA with a double-blind crossover design [30]. They found approx. 50% improvement in naming trained items after tDCS compared with 20% improvement during therapy combined with sham stimulation immediately after 10 days of stimulation. At follow-up two weeks after the end of therapy, the two svPPA patients showed only a 7% decrease in naming trained items. In another study, three patients with svPPA completed a modified SFA training combined with anodal tDCS to the left temporo-parietal cortex [31]. Two weeks after therapy, naming accuracy increased by an average of 20% for trained items, whereas it did not change for untrained items. Naming accuracy six months after stimulation decreased by 45% for trained items and by 60% for untrained items. Thus, no generalization effects of the modified SFA therapy in combination with tDCS on untrained items could be demonstrated. In addition, no conclusions could be drawn about the respective effects of the language therapy and tDCS, since no sham condition was included [31].

Taken together, compared to patients with other forms of PPA, individuals with svPPA appear to benefit less from speech and language therapy in terms of generalization to untrained items, as they progressively lose semantic knowledge and their semantic system is disrupted. A smaller effect of transcortical electrical stimulation on word retrieval and semantic knowledge as well as smaller long-term effects than in other PPA patients or no effects at all are observed. Thus, semantic-based therapy in combination with tDCS seems to be a promising method to maintain the language abilities of svPPA patients over a longer period of time. There was evidence of improvements in naming both trained and untrained objects, presumably by learning and applying a strategy to compensate for semantic constraints [20].

In a pilot study with the svPPA participant of the present study, we investigated whether anodal tDCS over the left anterior temporal region had an effect on her speech and language performance compared with sham stimulation [32]. The participant was treated with individualized language training in combination with tDCS or sham stimulation in an ABAB design (A = sham). This double-blind study showed that the participant was able to complete 25% more language tasks correctly during the weeks with concurrent tDCS stimulation than during the training weeks in which sham stimulation was administered [32]. From this pilot study, we concluded that tDCS had a significant impact on this participant’s language performance. However, the results also showed that semantic knowledge decreased slightly during the eight-month intervention, although access to the semantic system was still largely preserved. Three months elapsed between the end of this pilot study and the start of our current study. 

Therefore, the primary aim of the present study was to investigate whether personalized semantic feature analysis (SFA) with concurrent tDCS at the left temporal pole would lead to improvements in naming and comprehension in this participant. To this end, we conducted a longitudinal study with several follow-up tests over 14 months. We postulated that word retrieval would remain stable or even improve over this period due to the combined therapy, although literature reporting a 20–30% decline in performance over this period if no language and speech therapy is provided [33]. 

The second aim was to investigate whether generalization to untrained material could be achieved and whether these items would also benefit from the present therapeutic intervention. In addition, it was to be investigated how long a possible effect lasted after the training weeks.

The third objective was to investigate the time frame in which possible effects of this intervention can be detected and how long they last. Based on the evidence from the literature, we assumed that the effect should last at least 14 days [28,31]. 

The fourth objective was to examine whether SFA combined with tDCS had an effect on the participant’s connected speech and communicative skills. Some studies showed that the information content of language increased after training, e.g., [34], but others found no change, e.g., [35].

## 2. Method

### 2.1. Design

In a longitudinal single-subject design, the combination of SFA and tDCS was investigated over a period of 14 months.

At the beginning of the study, pre-testing with different neurolinguistic and neuropsychological test procedures was conducted. This was followed by four stimulation phases (S1–S4) in which SFA was performed simultaneously with tDCS on 10 consecutive working days. The duration of tDCS was 20 min and SFA therapy was approximately 30 min per day. Baseline testing was performed immediately before each stimulation phase, and 2–3 follow-up tests were performed immediately after, approx. two weeks after, and approx. four weeks after the end of stimulation (t_1_–t_3_). The intermediate tests were performed to verify how long any therapeutic effect was measurable. Short pauses took place between the stimulation phases. Some of the material used for therapy was developed in-house by an experienced clinical linguist or selected from existing databases. The picture-naming ability of trained and untrained items was tested, and the progression across stimulation phases was reviewed and compared. Two different item sets including nouns and verbs, respectively, were processed and presented randomly during therapy. At the end of the study, a post-testing was conducted, which included test procedures comparable to the pre-testing. There were 3.5 months between the pre-testing and the first stimulation phase S1. Also, there were 3.5 months between the last stimulation phase S4 and the post-testing. All testing appointments and therapy sessions were conducted at the participant’s home in a quiet environment, further reducing potential distractions. 

The study was approved by the Ethics Committee of Bielefeld University (2021-002) according to the German Psychological Society, the Professional Association of German Psychologists, and the Declaration of Helsinki. Written informed consent was obtained from both the participant and her husband before enrollment in the study.

### 2.2. Participant

The female participant was a 56-year-old right-handed native German speaker who was diagnosed with frontotemporal dementia (FTD) in 2016 by her treating neurologist. The participant had ten years of school education and completed vocational training as an industrial clerk. She is married and has a son. Everyday care tasks such as personal hygiene, household activities, grocery shopping, etc. were performed independently by the participant at the time of the study. In addition, she had an (adjusted) part-time job as an industrial clerk in a local company. 

The participant stated that she had noticed progressive memory problems, word-finding difficulties, problems with language comprehension at the one-word level, and concentration difficulties for about three years prior to diagnosis. There were no other known pre-existing conditions and no positive family history related to dementia. MRI revealed left accentuated bilateral temporal lobe atrophy and a small decrease in left hippocampal volume. PET-CT evaluation showed hypoperfusion in the left inferior lateral anterior and posterior temporal cortex. Microangiopathic lesions were mild and other intracranial findings were normal for the participant’s age. Other parameters such as general health and autonomic abilities were unremarkable. 

There were no other therapeutic interventions and the participant did not take any medications during the 14-month study period. For transcranial direct current stimulation, contraindications such as previous neurological diseases, epilepsy, epilepsy in close relatives, severe and frequent migraine, severe traumatic brain injury, presence of metal parts in the skull, surgery on the brain, chronic skin disease, and wearing a pacemaker or taking neuroactive medications were excluded [36]. 

Before the start of the study, the participant was informed verbally and in writing about the procedure and content of the study. The contraindications of tDCS, possible side effects, and data storage were also explained in this way. The participant confirmed in writing that participation in the study was voluntary and that consent could be withdrawn at any time without giving reasons. Further questions could be asked at any time during the informative interview.

### 2.3. Neurolinguistic and Neuropsychological Assessment

Before and after the therapeutic intervention, various language and cognitive tests were administered (pre-testing/post-testing). The selected diagnostic instruments were largely based on the established diagnostic criteria for svPPA [1] (Table 1). 

The test procedures for assessing the participant’s cognitive performance and speech and language production included an analysis of the spontaneous speech, which is based on the criteria of the Aachen Aphasia Test (AAT) [37]. The topics selected by the therapist were close to the participant’s everyday life in order to stimulate guided spontaneous speech. In the pre-testing, the topic of traveling was discussed, as this was a hobby of the participant. The topics of the spontaneous speech in the post-testing were food, travel, and both general and personal changes due to the COVID-19 pandemic. The spontaneous speech analysis was performed by the investigator at the beginning of the diagnostic session to give the participant a casual start. The conversation was recorded with a recording device (Olympus, Tokyo, Japan, VN-731PC) and later transcribed and analyzed manually. Further, the Boston Naming Test for confrontation naming (BNT; [38]); the Bogenhausen Semantics Examination, a predominantly nonverbal semantic test for object knowledge (BOSU; [39]); the TROG-D [40], a test of grammar function and single-word comprehension; the Regensburg Word Fluency Test (RWT; [41]); and the repetition subtest of the Aachen Aphasia Test (AAT; [37]) were performed. In addition, verbal memory and general cognitive abilities were assessed with a verbal learning and memory test (VLMT; [42]), the DemTect (version A) [43], and the Montreal Cognitive Assessment (MoCA; [44]). Further, the Geriatric Depression Scale (GDS; [45]) was applied (results see Table 1).

### 2.4. Therapy Material

**Figure 1:**
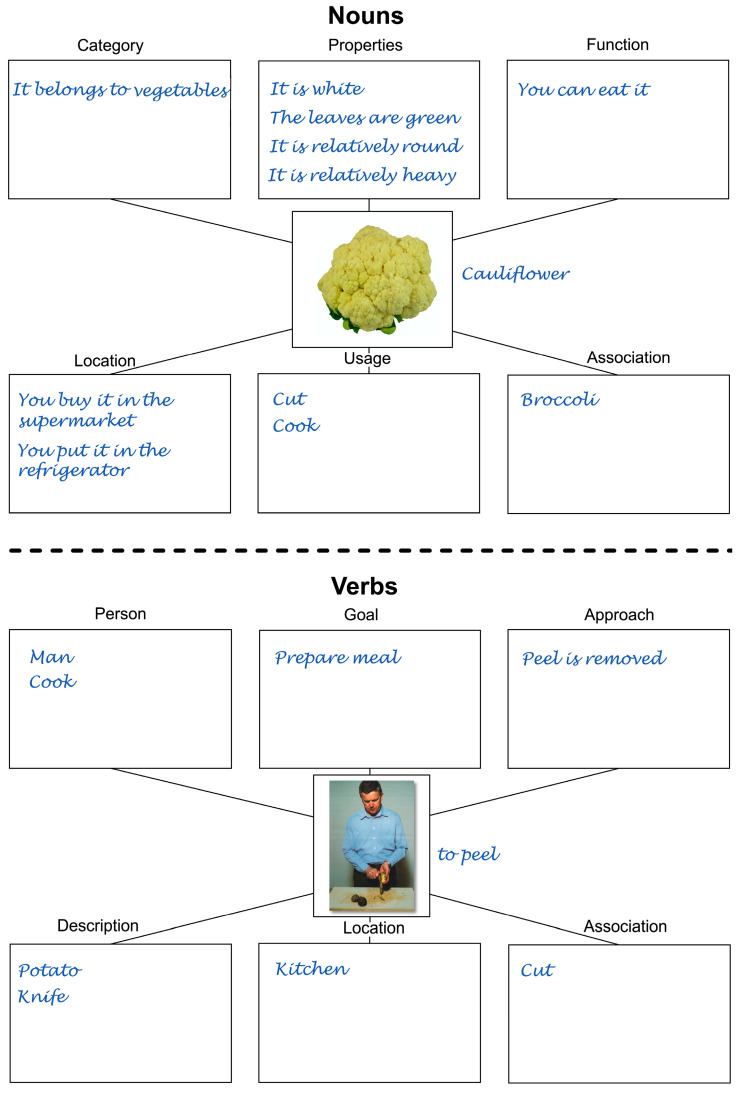
SFA sheets for nouns and verbs. (**top**): SFA sheet for nouns for the word “Blumenkohl” (*cauliflower*) as an example. (**bottom**): SFA sheet for verbs for the word “schälen” (*to peel*) as an example. The participant’s original responses were translated into English.

Various worksheets for nouns and verbs, respectively, were created for the therapy (Figure 1). These were based on a template used in a single case study [46]. A separate worksheet was used for each item. The goal of the therapy was to fill in all semantic fields of the worksheets in writing in order to be able to name the item during processing or at the end of processing. The participant could decide which fields to fill in first. In case of difficulties with the semantic fields, the therapist provided assistance. Regarding the nouns, first, the category to which an object belongs was asked, then the properties of an object, the place where a certain object can be used or purchased, the usage of an object, and a spontaneous association to an object similar to the object depicted (Figure 1, top).

The worksheet used for processing the verbs was similar in structure to that used for the nouns. The semantic fields were based on a template [47]. First, the participant had to identify the person performing the action, and then the goal pursued by the action (Figure 1, bottom). It was also possible to mention previous or further actions that were directly related to the action presented. Then, the location where the action takes place and an association had to be described. Finally, the target word for nouns and verbs should be written down at the lower right margin of the sheet.

The visual material for processing nouns was taken from the Bank of Standardized Stimuli (BOSS) which consists of colored photographs that can be divided into 23 semantic categories [48,49]. The nouns used can be assigned to the semantic categories of animals, food, household items, office supplies, furniture, electrical items, clothing, and tools. The nouns used were divided into two lists, each used during stimulation phases S1 and S3 and during stimulation phases S2 and S4, respectively. Each list consisted of 40 words, which were tested at the beginning of the stimulation phases during the baseline survey. The average word frequency of the nouns of set 1 (M = 1152.54, SD = 4473.37) and set 2 (M = 601.74, SD = 1212.99) was statistically equivalent (*t* = 1.47, *p* = 0.14). The following psycholinguistic parameters were defined using five-point Likert scales [49]. In list 1, the selected items had a high familiarity score (M = 4.36, SD = 0.56), as did those in list 2 (M = 4.24, SD = 0.68). No significant differences were found (*t* = 1.10, *p* = 0.27). In terms of visual complexity average values for list 1 (M = 2.45, SD = 0.56) and list 2 (M = 2.47, SD =0.65) were obtained, but no significant differences were found (*t* = 0.96, *p* = 0.34). Moderately high values were obtained for object agreement in both set 1 (M = 3.85, SD = 1.31) and set 2 (M = 3.91, SD = 1.81) with no significant differences between the two sets (*t* = 0.62, *p* = 0.53). For viewpoint agreement, moderately high values were achieved for set 1 (M = 3.65, SD = 1.24) and set 2 (M = 3.79, SD = 1.21), with no significant differences found (*t* = 1.26, *p* = 0.21). Furthermore, the manipulability was examined for set 1 (M = 2.8, SD = 0.79) and set 2 (M = 2.65, SD = 0.88). Again, no significant difference was found between sets 1 and 2 for this parameter (*t* = 0.29, *p* = 0.77). The last parameter studied was the number of syllables in the words of set 1 (M = 2.08, SD = 0.88) and set 2 (M = 2.56, SD = 0.89). A significant difference was found for the word sets (*t* = −2.81, *p* = 0.005).

The visual material for verbs was taken from the Everyday Life Activities (ELA) item set [50]. In the present study, ELA set 1 was used. This set shows the actions of a family’s everyday life. These everyday activities are divided into different semantic fields which include gestures and facial expressions, movements and postures, daily physical needs, dressing, food and drink preparations, eating and drinking, household activities, shopping, necessary daily actions, social interactions, leisure, hobbies, and sports [51]. The verbs used were all full verbs and concretes. The verbs represented activities in the kitchen and in the household, with tools, sports and exercise, leisure activities, music, and emotions.

Verbs were also divided into two lists. The first list was used during stimulation phases S1 and S3, and the second list was used during S2 and S4. As with nouns, each list consisted of 40 words that were tested at the beginning of a stimulation phase in the baseline survey. For verbs, two-tailed Student’s *t*-tests for independent measures were performed, too. For the average word frequency in set 1 (M = 3025.16, SD = 6755.79) and set 2 (M = 619.79, SD = 2044.44), no significant difference was found (*t* = 1.97, *p* = 0.051). The average syllable number in set 1 (M = 2.2, SD = 0.57) and set 2 (M = 2.4, SD = 0.75), was also not significantly different (*t* = −1.01, *p* = 0.31).

### 2.5. tDCS Equipment

**Figure 2:**
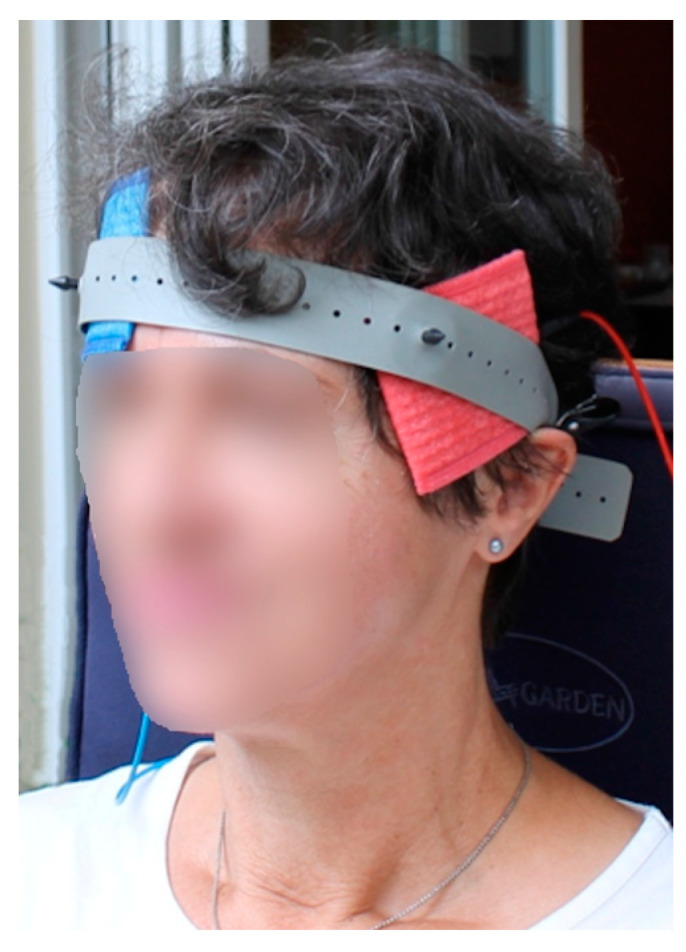
tDCS application. Placement of the tDCS electrodes: anode (red) over the left temporal pole, cathode (blue) at the right supraorbital region.

Transcranial current stimulation was performed using a battery-powered direct current stimulator (neuroConn, Ilmenau, Germany). Stimulation was performed using two conductive rubber electrodes (5 × 7 cm^2^) placed in sponges soaked in 0.9% NaCl solution. Before placing the electrodes, the corresponding skin sites were gently rubbed with a cotton swab soaked in 70% alcohol to improve impedance. The average value of impedance was 3.9 kOhm at the beginning of stimulation, 3.1 kOhm after 10 min of stimulation, and 3.0 kOhm toward the end of stimulation. Placement of the electrodes was performed according to the international 10-10 EEG system. The anode was placed on the intersection between FT7 and FT9, with the long side of the sponge electrode oriented horizontally and slightly inclined next to the left ear (Figure 2). This electrode position is located approximately over the left anterior temporal lobe and has already been used successfully in a previous study [28]. The cathode was placed on the contralateral side on the supraorbital region (Fp2), horizontally aligned with the long side of the sponge electrode. During the experiment, anodal tDCS was applied for 20 min with a current intensity of 1.5 mA, and fade-in and fade-out phases of 10 s each. Based on the results of the pilot study and the participant’s personal request, no sham stimulation was performed in this study to provide the participant with the optimal intervention over the 14 months. tDCS was performed concurrently with the training phases with SFA. The subject cooperated well and her attention and concentration remained stable during the sessions. During the intermediate tests, when no tDCS was performed, the participant needed more motivation toward the end of the study. This was always achieved by positive verbal reinforcement by the therapist.

The participant’s performance in all stimulation phases and test sessions was recorded with an audio recorder (Olympus, Tokyo, Japan, VN-731PC).

### 2.6. Experimental Procedure

**Figure 3:**
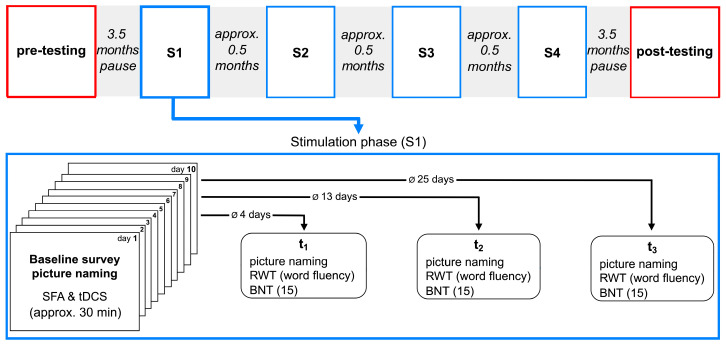
Experimental procedure. Flow chart of the stimulation phases S1–S4. For S1, the detailed sequence of the intervention is shown as an example.

A diagnostic pre-testing was performed prior to the therapeutic intervention (Figure 3). Further, at the beginning of each stimulation phase (S1–S4), a baseline survey took place in which the participant was asked to name 40 nouns and 40 verbs shown in the form of picture cards. Afterward, SFA training and concurrent tDCS started.

The items that were not named correctly in the baseline survey were trained with the combination of SFA and tDCS for ten consecutive working days in each stimulation phase. On average, 24 items were practiced per stimulation phase. The stimulation lasted 20 min and therapy in total of 30 min per day. After ten days of therapy, intermediate tests (t_1_, t_2_, t_3_) were performed. In each stimulation phase, the intermediate tests took place approximately 4, 13, and 25 days after the last stimulation session. During the intermediate tests, a picture naming task, four subtests of the RWT, and the 15-item version of the BNT were administered. If a 10% drop in performance was detected on at least one of the tests, the next stimulation phase was initiated. In the stimulation phase S3, only two intermediate tests were performed because a drop in performance of more than 10% in picture naming was found in the interim test t_2_ when the trained items were examined. Therefore, t_3_ was omitted in this stimulation phase. In addition, there were pauses of about 0.5 months between all stimulation phases due to organizational reasons.

The intermediate tests took place without tDCS. The picture naming test consisted of the noun and verb material which was used in the baseline survey (untrained words) and in the therapy (trained words). During this test, the participant did not receive any feedback as to whether she had named an item correctly or not. In addition to the words from the baseline survey, the untrained words consisted of words that the participant had not seen before. The words in the baseline surveys and the previously 30 unseen untrained words were identical in S1 and S3 and S2 and S4, respectively. The naming performance of the trained and untrained items was analyzed separately. The four subtests of the RWT each consisted of a semantic fluency task, a semantic category-switching task, a formal lexical fluency task, and a formal lexical category-switching task. The shortened version of the BNT was used as a standardized procedure for naming performance. At the end of the entire intervention, a post-testing was performed (Figure 3). 

Pre-testing was performed 3.5 months before the start of the first stimulation phase S1 on two consecutive working days. The three and a half-month break between diagnostic pre-testing and S1 was due to the participants’ work and leisure activities, which had to be taken into account when planning the study. The tests on both pre-testing days lasted about two hours and were adapted to the participant’s ability to concentrate and pay attention. It is not to be expected that the result of the diagnostics with regard to the clinical picture would have changed during this period. However, before each stimulation phase, a baseline for picture naming was obtained, which served as a reference for a possible improvement due to the stimulation unit.

The post-testing also took place 3.5 months after the end of the last stimulation phase S4 and was also performed on two consecutive working days. The delay between the last stimulation phase and the post-testing was due to the COVID-19 pandemic and contact restrictions.

Post-testing also took approximately two hours per day. The participant had the opportunity to request a break at any time during the tests. The whole period between pre- and post-testing was 14 months.

### 2.7. Data Analysis

The data collected were statistically analyzed using Jamovi (vers. 2.3; [52]). The statistical significance level was set at 5% (α = 0.05) for all analyses. The *p*-values were Bonferroni corrected where necessary. Binominal logistic regression was used to investigate whether the participant’s picture naming performance improved after the intervention. First, we investigated whether word type (nouns vs. verbs) or the word lists used predicted whether an item was named correctly or not. Second, we analyzed whether the training status of the words in each set (trained vs. untrained) predicted naming performance. No feedback was given to the patient during the testing to avoid learning via the tests. Furthermore, we tested whether the individual test time points t_1_ to t_3_ of the intermediate results predicted better naming performance of the untrained items to investigate generalization effects. This was done for each single phase (S1–S4) and further consolidated for all four intervention blocks.

Paired *t*-tests were used to compare the item sets concerning their linguistic parameters. The pre- and post-testing conducted at the beginning and the end of the study were analyzed descriptively. In addition, linear regression analyses were used to compare the data from the BNT as well as the RWT administered during pre-testing, post-testing, and interim-testing.

## 3. Results

### 3.1. Behavioral Results of Pre- and Post-Testing

The participant was examined and diagnosed by an experienced clinical linguist before and after the therapeutic intervention; the time between the pre- and post-tests was 14 months. A series of neurolinguistic and neuropsychological tests were performed and the results, norm scores, and criteria for interpretation of the test results are listed in Table 1.

When analyzing spontaneous speech, the ratio of content words to phrases was 1.8 in both the pre-test and post-test, with an average of 6.2 words produced per sentence in the pre-test and 6.0 words per sentence in the post-test. This indicates that speech continued to be fluent. Speech rate, averaging 108 words per minute at pre-testing and 107 words at post-testing, could also be considered normal. The phrases were characterized by a short and simple syntactic structure with a stringing together of main clauses and few subordinate clauses. Strikingly, the analyses of the spontaneous speech of the pre- and post-testing revealed clear differences in the number of word-finding difficulties (Table 1). While the ratio of word-finding difficulties per phrase in the pre-testing was 0.32, the value in the post-testing was 0.19, reflecting an improvement of more than 40%. This indicates a possible effect of the therapeutic intervention on the single-word level in spontaneous speech and thus on the participant’s communicative abilities. All in all, at the time of pre- and post-testing, the language abnormalities identified during standardized tests could still be compensated well by the participant in spontaneous speech.

Naming performance in the BNT showed significant performance deficits both before and after the intervention (Table 1). During the pre-testing, the patient could spontaneously name 25% of the items correctly. After semantic cues, a total of approx. 28% of the items were named correctly. After phonematic cues, no item could be named correctly. During post-testing, 17% of the items were spontaneously named correctly. After the semantic cues, the participant could not name a further item correctly. However, she was able to correctly name an additional 33 items after the phonematic cues, so she was able to correctly name a total of 75% of the items in the post-testing.

The BOSU examined functional and associative semantic memory performance with five subtests. In four of the five subtests, the participant showed a significant decrease in the ability to assess semantic relations at the post-testing. In particular, situational semantic relations or the hierarchy of semantic knowledge was increasingly affected over the 14-month study period.

The TROG-D tested grammar comprehension using 21 subtests. It was found that the subject’s grammatical skills were comparatively unaffected and had even improved minimally over the course of the therapeutic intervention. The errors that occurred were all lexical in nature, which could indicate deficits in single-word and sentence comprehension.

The RWT was used to examine semantic and lexical word fluency as well as word fluency during a semantic and lexical category change. A testing time of two minutes per subtest was chosen both for pre-testing and post-testing. The results were well below the average word fluency, corresponding to a percentile rank of ≥10. At post-testing, the impairment was even more pronounced.

The ability to repeat was tested using the “Repeat” subtest of the AAT. Sounds, monosyllables, loan- and foreign words, compound words, and sentences were tested. The participant achieved a score of 146 (out of 150), which corresponds to a percentile rank of 94. This test was administered only during the pre-testing and the participant did not show any deficits in repeating.

All in all, the language test procedures make it clear that the subject’s difficulties were primarily in word retrieval with visual stimuli and in word fluency.

The VLMT showed slightly decreased memory performance in verbal learning, recall, and recognition which became more pronounced in the post-testing. Both the DemTect and the MoCA showed a mild cognitive impairment of the participant already during the pre-testing, with scores worsening during the post-testing. In the performance of the MoCA, the conspicuities were particularly in the repetition of sentences (main and subordinate clauses), in the word fluency task, and in the delayed word recall of a word list. During the post-testing, short-term memory (repeating number sequences backward) and semantic abstraction (naming the commonality of two concepts) additionally deteriorated. In the DemTect, the word fluency task was especially conspicuous during pre-testing. During post-testing, a deterioration in the ability to convert numbers was also observed. The GDS indicated mild to moderate depression only at the post-testing.

Overall, neurolinguistic and neuropsychological testing revealed semantic deficits in this participant in terms of impaired single-word comprehension of low-frequency words and impaired object-naming skills. Thus, the two mandatory criteria for the diagnosis of svPPA were met [1]. Furthermore, spontaneous speech was characterized by well-preserved surface structure and fluency. The BOSU showed difficulty with object knowledge. In contrast, language comprehension at the sentence level and repetition of verbal items were virtually unimpaired. From these findings, it can be inferred that at the beginning of the therapeutic intervention, the participant had svPPA in an early stage since word-finding difficulties and difficulties in single-word comprehension were the most prominent impairments, while other linguistic domains were relatively intact. Also, no changes in behavior were noted that would indicate a later stage of svPPA [53]. In addition, decreases in other semantic abilities were noted in functional and associative semantic knowledge, semantic and formal lexical word fluency, and word retrieval. The cognitive screening indicated a mild cognitive impairment which became more severe over the course of the study. All in all, the participant showed no obvious impairments in the areas of phonology, syntax, grammar comprehension, and repetition at the time of therapeutic intervention. This finding indicates intact speech and language skills outside of (lexical-) semantic processing.

### 3.2. Results of SFA in Combination with tDCS

A total of 25 items (12 nouns, 13 verbs) were processed in the stimulation phase S1, 24 items (12 nouns, 12 verbs) in S2, 23 items (11 nouns, 12 verbs) in S3, and a total of 24 items (11 nouns, 13 verbs) in S4.

First, we investigated whether word type (nouns vs. verbs) predicted whether an item was named correctly or not. Binominal logistic regression showed no effect of word type (B = 0.15, SE = 0.12, *Z* = 1.2, *p* = 0.22). Therefore, the words were considered as one group in the following analyses. A further question concerned the two word lists used in the different stimulation phases. The word list did not predict whether an item was named correctly or not (B = 0.04, SE = 0.12, *Z* = 0.34, *p* = 0.73). However, there was a significant effect of training on the correctness of naming. More trained items were named correctly than untrained items (B = 0.82, SE = 0.13, *Z* = 6.14, *p* < 0.001).

**Figure 4:**
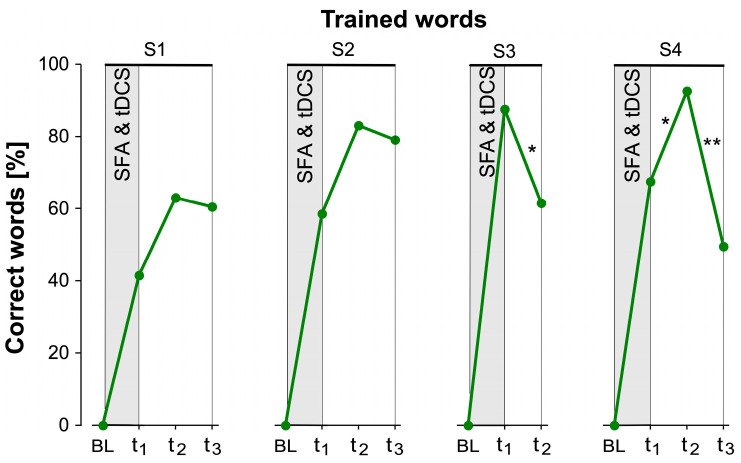
Results for trained words. Percentage of correctly named trained words in the baseline (BL), and the intermediate testing time points t_1_, t_2_, and t_3_ for all four stimulation phases. t_1_ took place on average 4 days after the simultaneous SFA and tDCS therapeutic intervention (SFA & tDCS), t_2_ after about 2 weeks, and t_3_ after about another 4 weeks. *p*-values are Bonferroni corrected (* = *p* < 0.05, ** = *p* < 0.01).

Another aspect addresses the question of which of the respective test time points within a stimulation phase were predictors for the correct or incorrect naming of items in the other test time points. For this purpose, binomial logistic regression was performed for each test time point separately for trained and untrained words. The results for trained items indicate that the ability to retrieve trained words actually increased until two weeks after the end of stimulation (Figure 4). In S4, this was significant (B = 1.705, SE = 0.856, *Z* = 1.99, *p* = 0.05). In S3, naming performance already decreased significantly between t_1_ and t_2_ (B = −1.455, SE = 0.752, *Z* = −1.94, *p* = 0.05). In S4, it decreased significantly only between t_2_ and t_3_ (approximately 2–4 weeks after the intervention) (B = −2.398, SE = 0.844, *Z* = −2.84, *p* = 0.004). Figure 4 shows the percentage of correctly named trained words for all stimulation phases and intermediate tests. In the baseline (BL), none of the subsequently trained words could be retrieved. The percentage of correctly named words increased by approximately 20% from t_1_ to t_2_ in all stimulation phases except S3. 

Binominal linear regression for untrained words showed that the participant’s word retrieval ability changed significantly in S2, S3, and S4 (Table 2). Compared to the baseline, S2 showed significantly higher naming performance in all three intermediate tests. In S3, naming performance increased significantly from BL to t_1_. In S4, naming performance increased significantly and decreased significantly again from t_2_ to t_3_ (Table 2).

**Figure 5:**
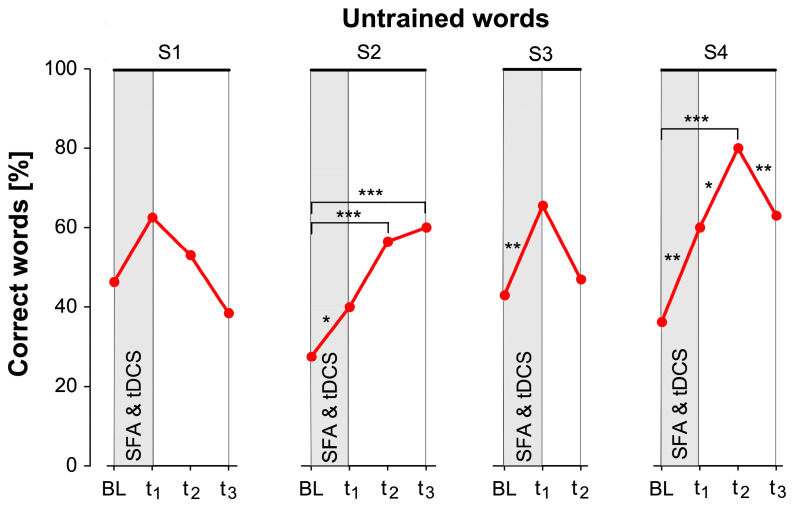
Results for untrained words. Percentage of correctly named untrained words in the baseline (BL), and the intermediate testing time points t_1_, t_2_, and t_3_ for all four stimulation phases. t_1_ took place on average 4 days after the simultaneous SFA and tDCS therapeutic intervention (SFA & tDCS), t_2_ after about 2 weeks, and t_3_ after about another 4 weeks. *p*-values are Bonferroni corrected (* = *p* < 0.05, ** = *p* < 0.01, *** = *p* < 0.001).

For the untrained items, naming performance increased by about 20% on average in all simulation phases, from baseline to t_1_ (Figure 5). In S2 and S4, performance increased again by about 20% in t_2_. In t_2_ of S4, the participant was even able to retrieve 80% of the untrained items correctly, which corresponds to an increase of about 40% compared to the baseline.

It is also interesting to examine how the participant’s naming performance changed at each intermediate test, averaged over all four stimulation phases, to obtain an overview of what the intervention did overall and how long the performance improvement lasted on average for this subject.

**Figure 6:**
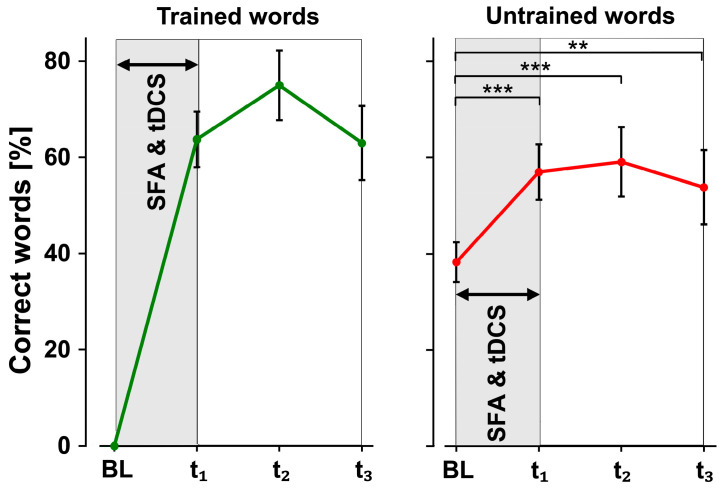
Averaged results for trained and untrained words. Mean percentage of correctly named trained (**left**) and untrained words (**right**) at the four test times (baseline, t_1_, t_2_, t_3_) averaged across all stimulation phases. SFA & tDCS means simultaneous therapeutic intervention. *p*-values are Bonferroni corrected (** = *p* < 0.01, *** = *p* < 0.001).

For the trained words, binomial logistic regression revealed no significant changes between each averaged test time point. On average, naming improved from the baseline by 60% in t_1_ and by an average of 75% in t_2_ after therapy compared to baseline values (Figure 6, left).

For the untrained words, binomial logistic regression showed that significantly more words could be correctly named at all three test time points compared to the baseline (t_1_: B = 0.800, SE = 0.200, *Z* = 3.99, *p* < 0.001; t_2_: B = 1.021, SE = 0.206, *Z* = 4.95, *p* < 0.001; t_3_: B = 0.648, SE = 0.218, *Z* = 2.98, *p* = 0.003). Again, there were no significant changes between test time points (Figure 6, right). These results indicate that the participant was able to transfer the learned strategies of SFA to the untrained words. These changes persisted for at least up to 4 weeks, with a peak two weeks after the end of the intervention.

### 3.3. Results of the BNT

**Figure 7:**
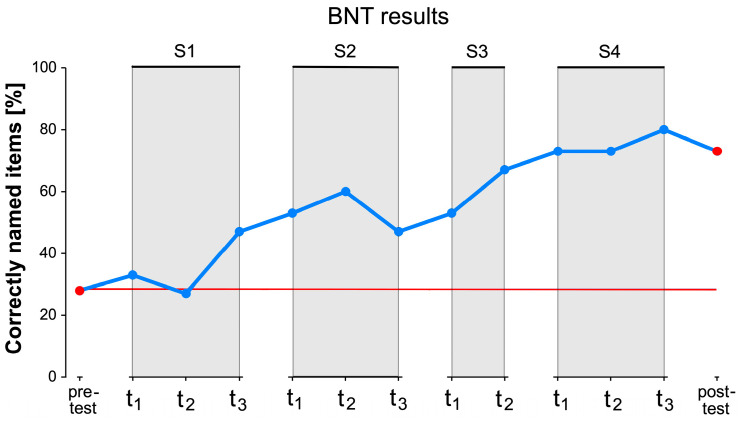
Results of the Boston Naming Test. Percentage of all correctly named items in the BNT in pre-testing, post-testing, and all intermediate tests for all stimulation phases, regardless of whether correct spontaneously or after given cues (blue line). The red line represents the initial percentage of correctly named items in the pre-test over the entire period of the intervention and illustrates in particular the difference between the results of the pre- and post-test.

A standardized test procedure for naming at all test time points was the BNT. In the intermediate tests, 15 of the 60 items were tested. The versions of the shortened BNT were identical in stimulation phases S1 and S3 and in stimulation phases S2 and S4. BNT results showed an increase from pre-testing to post-testing. In pre-testing, about 30% of the items were correctly named; in post-testing, about 70% (Figure 7).

Linear regression showed that the stimulation phase predicted the number of correctly retrieved items independent of cues (R^2^ = 0.93). There were significant results for the second stimulation phase (S2) compared to S1 (B = 3.33, SE = 0.713, *t* = 4.68, *p* = 0.014), for S3 compared to S1 (B = 4.33, SE = 0.797, *t* = 5.44, *p* = 0.006), and for S4 compared to S1 (B = 6.67, SE = 0.713, *t* = 9.35, *p* < 0.001). Further, S4 predicted a higher number of correctly named items than S2 (B = 3.33, SE = 0.713, *t* = 4.68, *p* = 0.014). All *p*-values were Bonferroni corrected. Strikingly, after a semantic cue, the participant was unable to correctly name an item at any of the intervening test time points. In contrast, the number of correctly retrieved items after a phonological cue increased with the number of phonological cues given (Pearson’s r = 0.66, *p* = 0.014).

### 3.4. Results of the RWT

**Figure 8:**
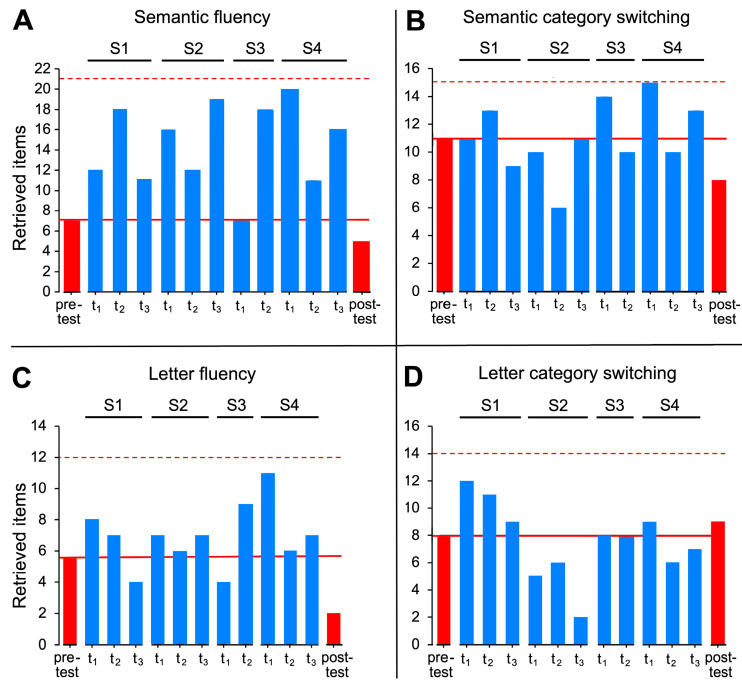
Results for word fluency. The absolute number of words retrieved in the semantic fluency, letter fluency tasks, semantic category switching, and letter switching tasks are shown separately for the pre-and post-testing (red columns) and the intermediate tests at t_1_, t_2_, and t_3_ of all stimulation phases (blue columns). The dashed red line represents age-appropriate norms, the solid red line represents the value collected in pre-testing.

The RWT, which tested semantic and lexical word fluency, switching between semantic categories, and switching between letters, was performed in the pre-testing, post-testing, and interim tests t_1_ to t_3_ of all stimulation phases. Compared to the pre-testing and post-testing, the probability of increased semantic word fluency averaged across all stimulation phases, only tended to be higher in t_1_, t_2_, and t_3_, but values were not significant after Bonferroni correction (Figure 8). For lexical fluency, only t_1_ showed a trend toward higher probability. Also, no significant predictions were found for semantic category switching and letter category switching (Figure 8). This means that the testing time points of the intermediate tests predicted performance in semantic fluency that improved slightly but not significantly in this participant compared to pre-testing but also post-testing. She improved two and five weeks after the intervention, although she still remained below the age-appropriate norm and showed decreased performance in the post-testing.

### 3.5. Results of the Comparison between First and Second Half of Intervention

In PPA there is often a difference in language in the first phase of a therapeutic intervention and the second phase [54]. We, therefore, compared the data on picture naming from the first half of the therapy (S1, S2) with those from the second half (S3, S4). We found no significant prediction of the intervention phase for the trained items (B = 0.175, SE = 0.223, *Z* = 0.786, *p*_bonferroni_ = 0.432). However, for the untrained items, we found a significant difference between the first and second half of the intervention (B = 0.362, SE = 0.15, *Z* = 2.44, *p*_bonferroni_ = 0.015; *exp* (*B*) = 0.695). The second half of the therapy resulted in a higher number of correctly named items. The BNT also showed significant results. In the second half of the therapy, significantly more items could be named (B = 4.07, SE = 1.078, *t* = 3.77, *p*_bonferroni_ = 0.004; *ŋ*^2^ = 0.613). No difference was found in the RWT between S1, S2, and S3, S4.

## 4. Discussion

In this longitudinal study over 14 months, the word retrieval performance of a participant diagnosed with svPPA was examined at several time points and in several tests. 

Prior to the study, the participant’s speech, language, and cognitive performance was assessed in a pre-testing using different neurolinguistic and neuropsychological tests. This was followed by four periods of therapy with SFA and simultaneous tDCS over 10 days. A baseline survey of picture naming was carried out before each therapy period, and 2–3 follow-up tests were carried out between the therapy phases. The start of a new therapy phase depended on whether the participant’s performance in word fluency and/or picture naming dropped by 10%. After the four therapy phases and all follow-up tests, a post-test using similar tests was carried out at the end.

The combined SFA/tDCS intervention resulted in improved word retrieval performance for the participant. The stimulation and therapy effects persisted for approx. two weeks after the end of each stimulation period. This was true for both trained and untrained words. Thus, a generalization effect could be measured. In addition, the number of word-finding difficulties decreased during guided spontaneous speech. The participant showed a significant effect of therapeutic intervention with the combination of SFA and tDCS. Compared to the baseline before the therapeutic intervention, she was able to retrieve about 60% more trained words correctly in the averaged first interim test. After about two weeks (t_2_), even 75% of the items could be named correctly. For the untrained items, an increase of about 20% was observed shortly after the stimulation phase (t_1_) compared to the baseline, and a further increase of about 5% was observed from t_1_ to t_2_. For both trained and untrained items, naming performance decreased again by an average of 10% from t_2_ to t_3_ (approx. 2–4 weeks after the end of the intervention).

### 4.1. Benefit of SFA

Previously, other authors demonstrated that SFA therapy per se is well suited to achieve an improvement in word retrieval in svPPA [5,7,21]. Through SFA, retrieval of concrete words in picture naming is facilitated by combining specific features and establishing connections between them. The elaboration of semantic concepts can also favor the generalization effect [15]. It has also been shown that therapy methods that have an additional conversational focus strengthen general communication skills [55]. The combined SFA and tDCS intervention may have an impact not only on single-word retrieval, but also on general communication skills, especially in spontaneous speech. 

### 4.2. Benefit of tDCS

Since it has been shown that word retrieval performance in svPPA in general decreases between 20 and 30% within a year the increase in performance of almost 15% in our study, especially in the untrained items, is remarkable [33]. In our study, SFA was combined with tDCS, because its effect on improved word retrieval for trained and untrained words was further enhanced by tDCS in several studies [20,28,29]. The ability to name pictures of trained items improved by 30% in svPPA patients after anodal tDCS of the left inferior parieto-temporal region compared to sham stimulation immediately after 10 days of stimulation [30]. tDCS is an effective addition to conventional speech and language therapy because the neural networks involved are enlarged by anodal electrical stimulation, making training effects easier to achieve [56]. Since in our study tDCS was administered on ten consecutive working days for 20 min each, we believe that this enhanced and prolonged the effects of SFA. The increase in BNT from the pre-test to the post-test (14 months) by about 40% also supports this assumption. In addition, some studies have shown that patients with PPA often show a difference in language performance between the first half of a therapeutic intervention and the second half [54]. In the present study, the participant was able to name significantly more untrained items correctly in the second phase of therapy and was also able to name more words correctly in the BNT. The significant increase in performance from the first half to the second half of the therapy could possibly have been achieved through the combined use of SFA and tDCS.

### 4.3. Untrained Items

The results with respect to the untrained items are particularly interesting. There was a significant increase in the naming of the untrained items compared to the baseline, up to two weeks after the end of the therapeutic intervention. Semantic-based therapy methods were found to produce significant learning effects for trained items, but improvements for untrained items were not demonstrated at significant levels [7,21]. To date, there is also limited evidence of generalization and maintenance effects on untrained items in patients with svPPA [12,15,16]. One explanation for the generalization of naming ability to untrained items could be that the participant learned the principles of SFA and applied them strategically when processing a new concept. Therapy methods that use self-cueing strategies favor generalization effects [14]. Another study showed that the elaboration of semantic concepts is more effective for word retrieval and repeated naming of items proved to be ineffective [57]. Another factor that influences generalization effects is item selection. The selection of meaningful words is important for retention and generalizability [5,15]. In our study, the trained and untrained words were chosen to correspond to a large extent to the participant’s personal life. Another aspect that may have positively influenced generalization is the fact that the participant was treated in a relatively early stage of the disease. Brain atrophy that is not yet far advanced and a relatively preserved semantic memory favors generalization effects [15]. In our view, a major factor that supported the increase in performance on the untrained words might also have been the combination of SFA and tDCS. Naming of untrained words has been shown to often improve after a combined intervention of speech and language therapy and transcranial brain stimulation [20]. tDCS can be considered an intervention that enhances therapy and generalization effects [58]. The increase in naming performance persisted for both trained and untrained words up to two weeks after the end of each stimulation phase. From this course, it could be concluded that stimulation periods at two-week intervals with short pauses were optimal for increasing the participant’s language performance. Similar to this study, the effect of improved language performance two weeks after the end of stimulation [29,31] and increased duration of treatment effects [58] have been demonstrated.

The BNT also showed that the number of untrained items that could be correctly named increased significantly over the course of the study. Interestingly, the number of words named spontaneously and without cues remained relatively constant during the intervention, but the number of words named correctly after phonological cues increased significantly. The number of words retrieved after phonemic cues correlated significantly with the number of phonemic cues. In addition, fewer semantic cues were required during the intervention. The participant was able to retrieve the target words by independently and adequately rewriting semantic connections in combination with phonological support. By combining semantic and phonological cues, the concept of a represented object could be more easily linked to the correct name [5,59].

### 4.4. Word Fluency 

A slight but not significant improvement in word fluency, as measured by the RWT was observed in the course of the 14 months. Semantic word fluency increased on average especially at t_2_ and t_3_ compared to pre- and post-testing. Phonological word fluency also tended to increase while letter switching and semantic category switching were comparatively stable. The slight improvement in word fluency during a stimulation phase could be due to the fact that the participant used self-directed cueing strategies, such as considering the semantic features of an object or activity. However, the age-appropriate norm values could not be reached at any test time point, so normalization of word fluency was not possible. The below-average performance in word fluency indicates that limitations in executive functions were already present at the time of the testing since word fluency tasks require cognitive flexibility.

### 4.5. Spontaneous Speech

A very promising trend emerged in the spontaneous speech analyses. While phonological, grammatical, and morphosyntactic features were relatively stable from pre- to post-testing, the number of word-finding difficulties decreased in the post-test 14 months later. A direct comparison of spontaneous speech showed that the number of word-finding difficulties decreased by 40%. This indicates that the participant was able to apply the compensation strategies of the SFA in spontaneous speech and thus in everyday communication. SFA can be considered a cueing strategy that simultaneously facilitates transfer to a specific context because it is universally transferable to the context used in everyday communication. Although studies have shown that word learning can generalize across trained items and that this can lead to improvement in everyday language, little research has investigated generalization across linguistic levels or tasks [7]. Some studies have reported that the information content of patients’ speech increases and that they show more correct information units in discourse after access to lexical items is facilitated [34]. However, others found no change in communication [35]. There is a possibility that the participant with svPPA in this study experienced improvements in connected speech because the effect of a conventional language therapy approach (SFA) was augmented with transcranial brain stimulation (tDCS). It has been shown that therapy methods with a conversational focus strengthen general communication skills. This effect might be enhanced by tDCS. Thus, an overarching goal of such therapy is not only to restore or improve language per se, but to prompt productive strategies to enhance existing communication skills [55].

Despite the significant improvements in naming ability for single words and spontaneous speech, a comparison of pre- and post-tests showed that participants’ cognitive abilities deteriorated over the course of the study. Thus, number processing ability, word formation, attention, and verbal memory decreased. Furthermore, there was a significant decline in semantic nonverbal abilities from the pre-test to post-test. Grammar comprehension and understanding of morphological syntactic structures remained largely intact. However, it is possible that the progressive course of svPPA would have developed more rapidly in the absence of language therapy combined with transcranial stimulation [33].

### 4.6. Possible Limitations

Although the results of our study are promising, certain limitations must be discussed. It cannot be differentiated whether the described findings would have occurred to this extent even without tDCS. In the present study, as already mentioned, a sham condition was deliberately omitted, because of its positive effect on the participant as a motivational factor and because she reported positive effects on her sleep, mood, general performance, and concentration. In addition, the pilot study showed significant improvements in language abilities with tDCS compared to sham [32]. Also, several studies demonstrated that the naming ability of trained, but especially of untrained words in patients with PPA can be improved in the long term by combining speech and language therapy with tDCS (for an overview see [20]). For these reasons, sham stimulation was ethically unacceptable to us.

Electrode positioning can also be debated. In most studies that investigated the effectiveness of tDCS in patients with PPA, stimulation was applied over left frontal areas [60,61,62]. In our study, we applied tDCS over the left anterior temporal areas, and thus over the brain areas affected by atrophy. However, significant effects were obtained in patients with svPPA with stimulation of the anterior temporal lobe and other studies also suggest a possible importance of stimulating the atrophied areas [23,28]. It has been found that for naming pictures of untrained items, only parieto-temporal tDCS yielded significantly higher scores after two months [29]. For the trained items, stimulation at both left inferior parieto-temporal sites and left dorsolateral prefrontal sites showed significantly greater improvement with real tDCS immediately at the end of the stimulation sessions. The parieto-temporal montage resulted in significantly better picture naming even two weeks after stimulation. This underlines the stimulation of left anterior temporal sites in our study.

## 5. Conclusions

In summary, although the current study is a single case study of a monolingual German native speaker, the results show a very promising possibility for the treatment of speech and language disorders in svPPA. Daily application of SFA combined with tDCS for ten consecutive working days in four stimulation phases improved naming ability for trained but also for untrained items in a participant with svPPA. We adapted the start of each new intervention phase to the patient’s performance in verbal fluency and picture naming and only started a new therapy phase when there was a drop of around 10% in one of the tests performed. Through this adaptive design, we found that stimulation intervals of two weeks were effective in counteracting the expected language and cognitive decline in this participant. The combined application of SFA and tDCS is a time-consuming and strenuous procedure for the patient. An interruption of the therapy of approx. 2–3 weeks showed no change in performance. The combined therapy may, therefore, not have to be continuous, but can take place at intervals. Most importantly, the linguistic compensation strategies of the SFA could be transferred to the participant’s everyday communication.

## Figures and Tables

**Table 1:** Neurolinguistic and neuropsychological diagnostics.

Test	Characteristics	Pre-Testing	Post-Testing
**Spontaneous** **speech**	minutes analyzed	9.03	7.37
	number of phrases	148	124
	content words	260	230
	set phrases	2	4
	stereotypes	9	5
	Word-finding difficulties	48	24
	echolalia	2	0
	sentence breaks	1	1
	missing sentence part	1	0
	sentence part duplications	0	2
	phonematic paraphasias	0	1
	syntactic structure	simple	simple
	speech production	fluent	fluent
			
**Test**	**Pre-testing**	**Post-testing**	**Norms**
**BNT**	Score	Score	
Items correct w/o assistance	15 */60	10 */60	55.73 (SD 4.42)
No. of semantic cues given	11/45	13/50	
No. correct following sem. cue	2/11	0/13	
No. of phonemic cues given	8/43	44/50	
No. correct following phon. cue	0/8	33/44	
Items correct with assistance	2/19	33/57	
**BOSU**	Raw value	Raw value	Cut-off-value
Assigning objects to situations	0	2 *	≥2
Sorting objects by main semantic features	0	0	≥2
Sorting objects according to semantic secondary features	0	2	≥3
Semantic sorting of written words	1 *	2 *	≥1
Sorting objects by color	0	1 *	≥1
**TROG-D**	Score	Score	
	18/21	19/21	
Grammatical errors	double object constructions topicalization relative clause	topicalization relative clause	
**RWT**	Percentile rank	Percentile rank	Percentile rank
Semantic fluency	<1 *	<1 *	≥10
Semantic category change	<2 *	<1 *	≥10
Lexical fluency	<3 *	<1 *	≥10
Lexical category change	<1 *	<1 *	≥10
**AAT (Repeat)**	Score		Score
	146		≥144
	Percentile rank		
	94		
**VLMT**	T-value	T-value	T-value
Learning	52	47	50 (SD 10)
Delayed recall	45	39 *	50 (SD 10)
Recognition	46	42	50 (SD 10)
**DemTect**	10 */18	9 */18	>13 points
**MoCA**	22 */30	20 */30	>26 points
**GDS**	3/15	7.5 */15	≤5 points

Neurolinguistic and neuropsychological tests administered before (pre-testing) and after the therapeutic intervention (post-testing). There were 14 months between the results of pre- and post-testing. Comparison of the results with the respective norm values. * Deviations from the (age-appropriate) norm data.

**Table 2:** Comparison of the intermediate tests for untrained words.

Stimulation Phase	Intermediate Tests	B	SE	*Z*	*p*
**S2**	BL-t_1_	0.825	0.393	2.10	0.036
	BL-t_2_	1.434	0.415	3.45	<0.001
	BL-t_3_	1.311	0.411	3.19	<0.001
**S3**	BL-t_1_	1.219	0.427	2.86	0.004
**S4**	BL-t_1_	1.096	0.406	2.70	0.007
	BL-t_2_	2.107	0.507	4.16	<0.001
	t_1_-t_2_	1.011	0.528	1.91	0.05
	t_2_-t_3_	−1.434	0.513	−2.80	0.005

Stimulation phases (S2, S3, S4) and test time points (BL, t_1_, t_2_, t_3_) predicting significant naming differences for untrained items. *p*-values are Bonferroni corrected.

## Data Availability

The data presented in this study are available on request from the corresponding author due to permission restriction.
